# Long-Term Patient-Reported Quality of Life After Stereotactic Body Radiation Therapy for Recurrent, Previously-Irradiated Head and Neck Cancer

**DOI:** 10.3389/fonc.2020.00083

**Published:** 2020-02-05

**Authors:** Joel Thomas, Hong Wang, David A. Clump, Robert L. Ferris, Umamaheswar Duvvuri, James Ohr, Dwight E. Heron

**Affiliations:** ^1^School of Medicine, University of Pittsburgh, Pittsburgh, PA, United States; ^2^Department of Radiation Oncology, School of Medicine, University of Pittsburgh, Pittsburgh, PA, United States; ^3^Department of Otolaryngology, Head and Neck Surgery, School of Medicine, University of Pittsburgh, Pittsburgh, PA, United States; ^4^Division of Hematology and Oncology, Department of Medicine, School of Medicine, University of Pittsburgh, Pittsburgh, PA, United States

**Keywords:** quality of life, SBRT (stereotactic body radiation therapy), head and neck cancer, disease recurrence, toxicity

## Abstract

**Objectives:** Long-term quality-of-life data following stereotactic body radiation therapy (SBRT) for recurrent head and neck cancer (rHNC) is underreported. We report patient-reported quality-of-life (PR-QOL) after at least 1 year post-treatment.

**Methods and Materials:** A retrospective review was performed on 64 patients receiving SBRT for previously-irradiated rHNC. PR-QOL was prospectively evaluated using the University of Washington Quality of Life Questionnaire. The mixed effects proportional odds model was used to assess post-treatment overall PR-QOL changes, as well as the effects of late toxicities, tumor volume > 25 cc, local failure, nodal recurrence, distant failure, prior neck dissection, performance status other than ECOG 0 or Karnofsky 100, sex, age >65, squamous vs. non-squamous primary histology, and specific organ recurrence.

**Results:** SBRT had no significant effect on overall PR-QOL at days 1-90 post-treatment (SBRT effect 0.035, *p* = 0.93) and days 91–365 (SBRT effect −0.30, *p* = 0.45). Beyond day 365, overall PR-QOL was significantly worse than baseline (SBRT effect −0.77, p =.03). Grade ≥3 late toxicities (*p* = 0.0072) and tumor volume > 25 cc (*p* = 0.032) predicted significantly worse overall PR-QOL. Oral cavity recurrence predicted significant decrements in chewing (*p* = 0.0006), swallowing (*p* = 0.0301), and taste PR-QOL (*p* = 0.02). Nasal recurrence predicted significant decrements in taste PR-QOL (*p* = 0.030). Grade ≥3 late dysphagia predicted significant decline in chewing (*p* = 0.039) and swallowing (*p* = 0.0004). Grade ≥3 late osteonecrosis predicted significant differences in pain PR-QOL (*p* = 0.0026).

**Conclusion:** PR-QOL across several domains declines immediately after SBRT for previously-irradiated rHNC before returning to baseline levels at 1 year. Long-term PR-QOL declines thereafter. Patients with grade ≥3 late toxicities or tumor volume >25 cc report reduced long-term overall PR-QOL, likely representing late disease progression. Specific organ recurrence and grade ≥3 late toxicities predict decrements in specific PR-QOL domains.

## Introduction

Locoregional failure remains a major challenge in the management of head and neck cancer, as it affects 20–50% of patients (1) and represents the most common cause of mortality (2). Surgical salvage offers the best outcomes for patients with recurrent disease, providing a median overall survival (OS) of 44 months, compared to 11 months in patients with unresectable disease (3). Most patients, however, are ineligible for definitive surgical resection due to medical co-morbidities and/or anatomical constraints (4). The remaining therapeutic options for patients with recurrent head and neck cancer (rHNC)—radiation therapy and/or systemic chemotherapy—have traditionally been suboptimal.

Stereotactic body radiation therapy (SBRT) has recently emerged as a promising salvage treatment for rHNC, as it provides similar clinical outcomes to intensity-modulated radiation therapy (IMRT) in a shorter treatment window of 1–2 weeks, compared to 6–7 weeks for IMRT, while potentially reducing toxicities (5). In a multi-institutional retrospective comparison of these two techniques, Vargo et al. reported acute toxicity rates of 16.6 and 11.7% for patients treated with IMRT and SBRT, respectively, with no significant difference in OS (6). However, there is a relative paucity of information on patient-reported quality-of-life (PR-QOL). Vargo et al. previously analyzed PR-QOL after SBRT ± cetuximab for rHNC with a median follow-up of 6 months. (7) They reported that overall PR-QOL, health-related PR-QOL, and specific domains—swallowing, speech, saliva, activity, and recreation—show gradual improvement after an initial decrement at 1-month post-treatment.

To our knowledge, there are no reports detailing the long-term PR-QOL after SBRT for rHNC. This is particularly relevant given the increasing OS in this cohort over the past two decades—postulated to be due to factors such as improved surgical care and the increasing prevalence of HPV-positive oropharyngeal cancer (8)—as well as reports of severe late toxicity rates after SBRT as high as 18.9% with long-term follow-up (9). Here, we report PR-QOL in a cohort of patients with rHNC treated with SBRT with a minimum post-treatment follow-up of at least 1 year. We aimed to identify potential predictors of PR-QOL, such as late toxicity.

## Materials and Methods

After institutional board review by the University of Pittsburgh Institutional Review Board, we retrospectively identified patients treated for locally-recurrent or second primary head and neck cancer between November 2004 and January 2016 who had received prior radiation and had at least 1 year of PR-QOL follow-up. We included patients with both squamous cell carcinoma of the head and neck and non-squamous histology because previous studies reported no significant difference in clinical outcomes following SBRT re-irradiation between these two patient populations (10). We excluded patients who received SBRT as a boost after primary definitive radiation therapy, as well as patients who received SBRT for metastasis from a primary tumor outside the head and neck.

### Treatment Characteristics

Patients received SBRT on alternating days over a 2-week period for a total of 5 fractions at a median dose of 44 Gy (IQR 40-44). Patients were treated using Cyberknife, Truebeam STx, and Trilogy treatment platforms. Techniques for contouring, patient setup, and treatment delivery have been previously described (11, 12). Patients receiving concurrent cetuximab received a loading dose of 400 mg/m^2^ on day−7 followed by 250 mg/m^2^ on days 0 and +8.

### Patient-Reported Quality-Of-Life

All patients prospectively completed the University of Washington Quality-of-Life-Revised (UW-QOL-R) questionnaire, starting in November 2004. The UW-QOL-R is a validated survey that uses a Likert scale to assess overall PR-QOL, health-related PR-QOL, and PR-QOL in multiple domains relevant to head and neck cancer patients (13). Patients are queried about their health and QOL over the 7 days preceding survey completion, and their responses can be further classified as physical (e.g., chewing, swallowing, speech, taste, saliva, and appearance), social/emotional (anxiety, mood, pain, activity, recreation, and shoulder function), or global (“health-related QOL compared to month before had cancer,” “health-related QOL during the past 7 days,” “overall QOL during the past 7 days”). Patients prospectively completed the surveys in clinic on days before and after initiation of SBRT.

When surveyed, patients choose among discrete categorical responses that are converted to ordinal values ranging from 0 to 100, with 100 indicating best QOL. For example, responses in the “chewing” domain include “I can chew as well as ever,” “I can eat soft solids but cannot chew some foods,” and “I cannot even chew soft solids,” which are converted to discrete ordinal values of 100, 50, and 0, respectively. Patients completed surveys at their initial consultations before treatment, at approximately 1 month post-treatment, and then approximately every 3 months thereafter.

### Statistical Analysis

All data analysis was performed with SAS 9.4 software (SAS Institute Inc, Cary, NC), SPSS Statistics 24 (IBM Corp., Armonk, New York) and RStudio Version 1.0.143. Acute and late toxicities related to SBRT treatment per the National Cancer Common Terminology Criteria for Adverse Events (CTCAE) Version 4 were retrospectively obtained per chart review. “Acute toxicity” referred to adverse events occurring within 3 months of SBRT initiation, whereas “late toxicity” referred to adverse events occurring >3 months of SBRT initiation. A *p-*value of <0.05 was defined as statistically significant. No adjustments were made for multiple comparisons in this exploratory study. Ten (16%) patients received more than one course of re-irradiation SBRT; the time intervals for these patients' post-treatment PR-QOL surveys were defined from the first course of SBRT re-irradiation.

Because the QOL endpoints were ordinal data, and most patients completed multiple PR-QOL surveys over time, the mixed effects proportional odds model was used to assess for effects of our hypothesized independent variables on PR-QOL. In this model, we assume that the logarithm of cumulative odds for each QOL value can be estimated by a linear function of the fixed effect(s) (i.e., our hypothesized variable), plus a patient-specific random effect (that is, patient ID is a random effect in the model). The effect of the hypothesized variable(s) is assumed to be the same for each cumulative QOL odds ratio; thus it is called a mixed effects proportional odds model (14). To illustrate, the hypothesized effect of independent variable “tumor volume > 25 cc” on our dependent variable “overall PR-QOL” may be assessed in this model by assuming that the logarithm of cumulative odds for each category of overall PR-QOL is a linear function of the indicator “tumor volume > 25 cc” and the time variable, plus their interaction. In this model, if the parameter estimate for the effect of “tumor volume > 25 cc” is significantly negative (provided that the interaction term is non-significant), that means a patient with “tumor volume > 25 cc” has significantly worse QOL, and vice versa for a significantly positive parameter estimate.

We divided the post-treatment time into 3 periods post-SBRT: days 1–90 (3 months), days 91–365 (4–12 months), and days 366 and beyond (1 year and later), and compared each interval with baseline PR-QOL using the mixed effects proportional odds model (as described above). Next, we assessed whether hypothesized variables predicted significant differences in baseline PR-QOL (using the two-sided Wilcoxon rank sum test) and post-SBRT PR-QOL (using the mixed effects proportional odds model). Specifically, we assessed the effects of the following variables on overall PR-QOL: late toxicities, tumor volume > 25 cc, local failure, nodal recurrence, distant failure, prior neck dissection, performance status other than Eastern Cooperative Oncology Group (ECOG) 0 or Karnofsky Performance Status (KPS) 100, sex, age > 65, and squamous vs. non-squamous primary histology.

We also assessed whether recurrence at specific organs predicted significant decrements in specific hypothesized PR-QOL domains. We tested the effect of oral cavity recurrence on chewing, swallowing, taste, speech; nasal recurrence on taste; and salivary gland recurrence on chewing, swallowing, taste, and saliva.

Next, we assessed whether specific late toxicities ≥ grade 3 predicted significant decrements in specific hypothesized PR-QOL domains. We tested the effect of dysphagia on chewing and swallowing; skin toxicity on appearance; xerostomia on saliva and taste; fatigue on activity and mood; and osteonecrosis on pain. Finally, we also assessed the temporal change in average value for each PR-QOL domain with respect to timing of SBRT treatment: baseline, as well as 1, 3, 6, 9, 12, 18, 24, 36, and 48 months after completion of SBRT. Specifically, we classified each UW-QOL-R survey by date of survey completion relative to date of SBRT completion (e.g., baseline, 1-month, etc.). Some time intervals contained multiple surveys by the same patient; to avoid biasing the time intervals with respect to those patients, we only included the completed survey whose time relative to SBRT completion was closest to the intended time intervals. For example, if a single unique patient had 2 surveys at 90 and 100 days post-SBRT, then we only included the “90 day” survey in the 3-month interval. Missing data were omitted when generating mean PR-QOL domain values for each time interval.

We also assessed temporal change in composite scores for “Physical Function” and “Social/Emotional Function” per UW-QOL-R guidelines (13). Specifically, a “Physical Function” score was generated as the simple average of domain scores from each completed survey with responses for at least 4 of the following: chewing, swallowing, taste, speech, appearance, saliva. A “Social/Emotional Function” score was generated as the simple average of domain scores from each completed survey with responses for at least 4 of the following: anxiety, mood, pain, shoulder, recreation, and activity.

## Results

From November 2004 to January 2016, 391 patients with previously-irradiated, locally-recurrent or second primary head and neck cancer were treated with SBRT. Three hundred (77%) of these patients completed the UW-QOL-R questionnaire at ≥1 time point. After exclusion of those with no PR-QOL follow-up past 1 year, 64 patients remained. Table 1 contains baseline characteristics for this cohort. The median age at treatment was 64 years (range: 32–88), with a gender distribution of 41 males (64%) and 23 females (36%). Median tumor size was 25 cc (range: 1–166 cc), and 43 (67%) patients had squamous histology. Thirty-three (52%) patients received concurrent biologic therapy, with 32 receiving cetuximab and 1 receiving pembrolizumab. Seventeen (27.0%) patients received prior neck dissection. The median prior dose of external beam radiation therapy was 66 Gy (range: 25–127 Gy), with a median elapsed time from prior radiation therapy to SBRT of 31 months (range: 3–249 months).

**Table 1:** Patient and treatment characteristics.

**Baseline Characteristics**	**Number (% or range) (*n* = 64)**
Median age (years)	64 (32–88)
Male	41 (64.1%)
Female	23 (35.9%)
Median tumor volume (cc)	25.3 (1–166)
**HISTOLOGY**	
Squamous cell	43 (67.2%)
Adenoid cystic	10 (15.6%)
Mucoepidermoid	2 (3.1%)
Myoepithelioma	2 (3.1%)
Salivary ductal carcinoma	2 (3.1%)
Acinic cell	1 (1.6%)
Adenocarcinoma	1 (1.6%)
Medullary thyroid carcinoma	1 (1.6%)
Melanoma	1 (1.6%)
Spindle cell	1 (1.6%)
Poorly differentiated/undifferentiated carcinoma	1 (1.6%)
**RECURRENCE SITE**	
Larynx/hypopharynx	4 (6.3%)
Nasopharynx	2 (3.1%)
Oropharynx	5 (7.8%)
Oral cavity	13 (20.3%)
Base of skull/nasal cavity/paranasal sinuses	15 (23.4%)
Salivary gland	10 (15.6%)
Lymph node	13 (20.3%)
Other	2 (3.1%)
Concurrent biologics	33 (51.6%)
**SETTING OF STEREOTACTIC BODY RADIATION THERAPY**	
Definitive	44 (68.7%)
Post-operative	20 (31.3%)
Prior neck dissection	17 (27.0%)
Median prior radiation dose (Gy)	66 (25–127)
Median re-irradiation interval (months)	31 (3–249)

**Figure 1:**
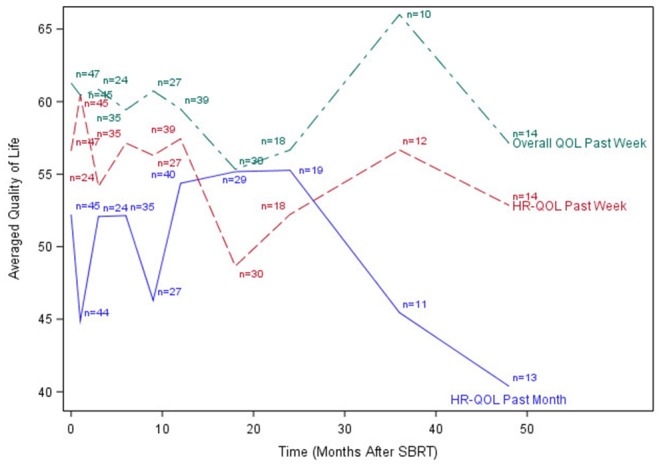
Post-treatment average patient-reported health-related quality-of-life and overall quality-of-life scores. HR-QOL, health-related quality-of-life; QOL, quality-of-life.

**Figure 2:**
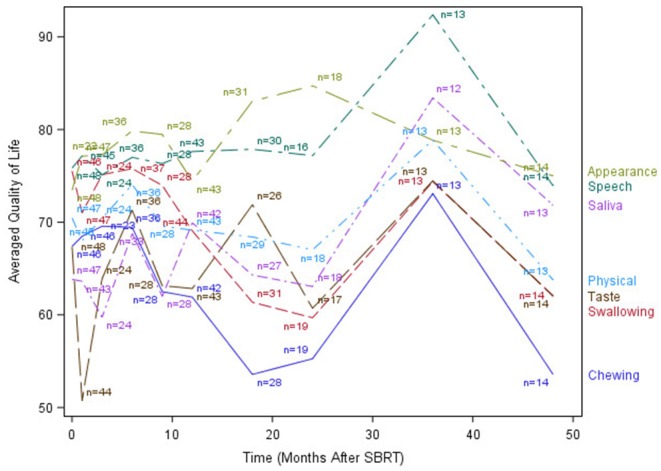
Post-treatment average patient-reported quality-of-life scores in chewing, swallowing, speech, taste, saliva, appearance, and Physical domains.

**Figure 3:**
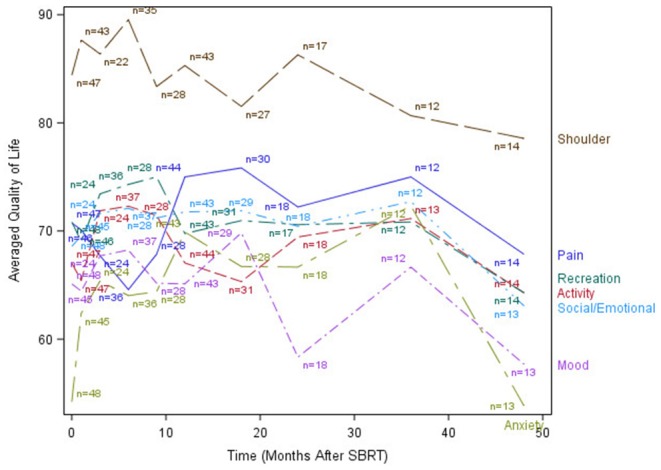
Post-treatment mean patient-reported quality-of-life scores in anxiety, mood, pain, activity, recreation, shoulder, and Social/Emotional domains.

The median time to last PR-QOL survey follow-up was 21 months (range: 12–107 months). The median number of surveys completed per patient was 5 (range: 1–10). Patients collectively completed a total of 305 surveys: 48 (75% of patients) at baseline, 47 (73%) at 1 month, 24 (38%) at 3 months, 37 (58%) at 6 months, 28 (44%) at 9 months, 44 (69%) at 12 months, 31 (48%) at 18 months, 19 (30%) at 24 months, 13 (20%) at 36 months, and 14 (22%) at 48 months. Figures 1–3 display the mean values for overall, health-related PR-QOL, and PR-QOL in multiple domains relevant to head and neck treatment, with respect to time after SBRT.

Per the mixed effects proportional odds model, post-SBRT overall PR-QOL was not significantly affected by time (time coefficient −0.00049, *p* = 0.12). Post-SBRT overall PR-QOL was not significantly different from baseline (SBRT effect −0.53, *p* = 0.10). In days 1–90 post-SBRT, treatment did not have a significant effect on overall PR-QOL (SBRT effect 0.035, *p* = 0.93). In days 91–365, SBRT did not have a significant effect on PR-QOL (SBRT effect −0.30, *p* = 0.45). However, beyond day 365, overall PR-QOL was significantly worse than baseline (SBRT effect −0.77, *p* =.03).

Table 2 reports the effects of hypothesized variables on overall PR-QOL. Patients experiencing grade ≥3 late toxicities had similar baseline overall PR-QOL (*p* = 0.34) but consistently lower overall PR-QOL after SBRT based on the mixed model. For instance, at initial follow-up post-SBRT, day 365 and day 730, the —values were, respectively, 0.0091, 0.0072, and 0.0059. Patients with tumor volume >25 cc had similar baseline overall PR-QOL (*p* = 0.067) but consistently lower overall PR-QOL after SBRT based on the mixed model. For instance, at initial follow-up post-SBRT, day 365 and day 730 the *p*-values were 0.029, 0.020, and 0.032, respectively. Local failure (*p* = 0.55), nodal recurrence (*p* = 0.61), distant failure (*p* = 0.48), prior neck dissection (*p* = 0.14), ECOG > 0 or KPS < 100 (*p* =.072), sex (*p* = 0.98), age >65 (*p* = 0.62), and non-squamous histology (*p* = 0.49) had no significant effect on PR-QOL at any time point after SBRT.

**Table 2:** Effects of patient variables on overall QOL Post-SBRT.

**Baseline Characteristics**					**Proportional Odds Model for Post-SBRT Median Overall QOL**			
**Group**	***n***	**Median Baseline QOL**	**IQR**	**WRST for Median Baseline Overall QOL *P-*Value**	**Initial follow-up Parameter estimate (*P-*Value)**	**Day 365 *P-*Value**	**Day 730 *P*-Value**	**Time Effect *P*-Value**
Grade ≥3 late toxicity	15	40	(40.0, 80.0)	0.3401	−1.79 (0.0091)	0.0072	0.0059	0.0870
No grade ≥3 late toxicity	31	60	(60.0, 80.0)					
Tumor volume > 25 cc	24	60	(40.0, 80.0)	0.0667	−1.43 (0.0285)	0.0197	0.0322	0.0901
Tumor volume ≤ 25 cc	19	60	(60.0, 80.0)					
Local failure	29	60	(40.0, 80.0)	0.6386	−0.39 (0.5492)	0.2801	0.1566	0.9783
No local failure	17	60	(40.0, 80.0)					
Lymph node recurrence	7	40	(40.0, 80.0)	0.4022	−0.42 (0.6140)	0.3243	0.1736	0.2723
No lymph node recurrence	40	60	(50.0, 80.0)					
Distant failure	15	60	(60.0, 80.0)	0.7974	0.48 (0.4829)	0.5777	0.7465	0.4298
No distant failure	30	60	(40.0, 80.0)					
Prior neck dissection	10	60	(60.0, 80.0)	0.4735	1.09 (0.1438)	0.3418	0.8220	0.5291
No prior neck dissection	37	60	(40.0, 80.0)					
ECOG 0 or KPS 100	11	60	(60.0, 80.0)	0.4619	1.48 (0.0722)	0.1034	0.2241	0.3929
Not ECOG 0 or KPS 100	26	60	(40.0, 80.0)					
Male	27	60	(40.0, 80.0)	0.6958	−0.020 (0.9753)	0.8739	0.7851	0.4834
Female	20	60	(40.0, 70.0)					
Age > 65	21	60	(60.0, 80.0)	0.6087	0.33 (0.6166)	0.4121	0.3001	0.1031
Age ≤ 65	26	60	(40.0, 80.0)					
Primary squamous histology	30	60	(40.0, 80.0)	0.5924	−0.47 (0.4901)	0.2806	0.1761	0.7362
Primary non-squamous histology	17	60	(40.0, 80.0)					

*QOL, Quality of life, IQR, Interquartile range, WRST, Wilcoxon Rank-Sum Test*.

Table 3 reports the effects of recurrence sites on specific PR-QOL domains. Patients with oral cavity recurrence had significantly different baseline chewing PR-QOL (*p* = 0.0041) but also consistently lower chewing PR-QOL after SBRT with significant decline over time (time effect *p* = 0.0006) based on the mixed model. For instance, at initial follow-up post-SBRT, day 365 and day 730 the p-values were 0.021, 0.024, and 0.037, respectively. These patients also had similar baseline swallowing PR-QOL (*p* = 0.19) but significantly lower swallowing PR-QOL at initial follow-up post-SBRT (*p* = 0.0301*)*. However, swallowing differences were non-significant at day 365 (*p* = 0.056) and 730 (*p* = 0.13), but swallowing PR-QOL did decrease significantly over time (*p* = 0.0002). These patients also had similar baseline taste PR-QOL (*p* = 0.51) but consistently lower taste PR-QOL after SBRT without significant decline over time (*p* = 0.41) based on the mixed model. For instance, at initial follow-up post-SBRT, day 365 and day 730 the *p*-values were, respectively, 0.02, 0.014, and 0.02. Oral cavity recurrence had no significant effect on speech at baseline (*p* = 0.48).

**Table 3:** Effects of Recurrence Site on Specific QOL Domains.

**Baseline Characteristics**					**Proportional Odds Model for Post-SBRT Median Overall QOL**			
**Group**	***n***	**Median Baseline Chewing**	**IQR**	***P*-value with the Wilcoxon Rank Sum Test**	**Initial follow-up Parameter estimate (*P*-Value)**	**Day 365 *P*-Value**	**Day 730 *P*-Value**	**Time Effect (*P*-Value)**
Oral cavity recurrence	12	50	(0.0, 50.0)	0.0041	−3.77 (0.0205)	0.0240	0.0365	−0.0021 (0.0006)
No oral cavity recurrence	34	100	(50.0, 100.0)					
Salivary gland recurrence	8	50	(50.0, 100.0)	0.9992	2.72 (0.1337)	0.1560	0.2257	−0.0018 (0.0006)
No salivary gland recurrence	38	50	(50.0, 100.0)					
**Group**	***n***	**Median Baseline Swallowing**	**IQR**	**P-value with the Wilcoxon Rank Sum Test**	**Initial follow-up Parameter estimate (*****P*** **Value)**	**Day 365** ***P*****-Value**	**Day 730** ***P*****-Value**	**Time Effect (*****P*****-Value)**
Oral cavity recurrence	12	67	(50.0, 100.0)	0.1910	−2.44 (0.0301)	.0557	0.1259	−0.0018 (0.0002)
No oral cavity recurrence	34	67	(67.0, 100.0)					
Salivary gland recurrence	8	67	(67.0, 100.0)	0.9887	2.06 (0.1182)	0.1557	0.2620	−0.0013 (0.0011)
No salivary gland recurrence	38	67	(67.0, 100.0)					
**Group**	***n***	**Median Baseline Taste**	**IQR**	**P-value with the Wilcoxon Rank Sum Test**	**Initial follow-up Parameter estimate (*****P*****-Value)**	**Day 365** ***P*****-Value**	**Day 730** ***P*****-Value**	**Time Effect (*****P*****-Value)**
Oral cavity recurrence	12	67	(50.0, 83.5)	0.5076	−1.89 (0.0189)	0.0142	0.0184	0.4060
No oral cavity recurrence	36	67	(33.0, 100.0)					
Nose recurrence	10	67	(67.0, 100.0)	0.5795	1.71 (0.0438)	0.0237	0.0296	0.4097
No nose recurrence	38	67	(33.0, 100.0)					
Salivary gland recurrence	8	100	(50.0, 100.0)	0.2507	1.27 (0.1496)	0.0580	0.0494	0.3442
No salivary gland recurrence	40	67	(33.0, 100.0)					
**Group**	***n***	**Median Baseline Speech**	**IQR**	***P*****-value with the Wilcoxon Rank Sum Test**	**Initial follow-up Parameter estimate (*****P*** **Value)**	**Day 365** ***P*****-Value**	**Day 730** ***P*****-Value**	**Time Effect (*****P*****-Value)**
Oral cavity recurrence	12	67	(67.0, 100.0)	0.3724	0.79 (0.4804)	0.2346	0.1092	0.1466
No oral cavity recurrence	36	67	(67.0, 100.0)					
**Group**	***n***	**Median Baseline Saliva**	**IQR**	***P*****-value with the Wilcoxon Rank Sum Test**	**Initial follow-up Parameter estimate (*****P*****-Value)**	**Day 365** ***P-*****Value**	**Day 730** ***P*****-Value**	**Time Effect (*****P*****-Value)**
Salivary gland recurrence	8	67	(50.0, 83.5)	0.9338	−0.18 (0.9114)	0.9741	0.9612	0.2993
No salivary gland recurrence	39	67	(33.0, 100.0)					

*QOL, Quality of life; IQR, Interquartile range; WRST, Wilcoxon Rank-Sum Test; ECOG, Eastern Cooperative Oncology Group; KPS, Karnofsky Performance Status*.

Patients with nasal recurrence had similar baseline taste PR-QOL (*p* = 0.58) but consistently lower chewing PR-QOL after SBRT without significant decline over time (*p* = 0.41) based on the mixed model. For instance, at initial follow-up post-SBRT, day 365 and day 730 the *p*-values were, respectively, 0.04, 0.024, and 0.030. Salivary gland recurrence did not have a significant effect on PR-QOL in chewing (*p* = 0.13), swallowing (*p* = 0.12), taste (*p* = 0.15), or saliva (*p* = 0.91).

Table 4 reports the effects of specific grade ≥3 late toxicities on PR-QOL domains. Patients with late dysphagia had similar baseline chewing PR-QOL (*p* = 0.37) and no significant differences post-treatment overall (*p* = 0.89) or at 1-year follow-up (*p* = 0.22). However, these patients experienced a significant decline in chewing at 2-years follow-up (*p* = 0.039). Both patients with (*p* = 0.0009) and without (*p* = 0.0013) late dysphagia experienced significant decline over time in chewing. These patients also had significantly worse baseline swallowing (*p* = 0.025) with consistently lower swallowing after SBRT with significant decline over time (*p* = 0.0004) based on the mixed model. For instance, at initial follow-up, day 365 and day 730 the *p*-values were, respectively, 0.019, 0.013, and 0.017.

**Table 4:** Effects of Grade ≥3 Late Toxicity on Specific QOL Domains.

**Baseline Characteristics**					**Proportional Odds Model for Post-SBRT Median Overall QOL**			
**Grade ≥3 Toxicity**	***n***	**Median Baseline Chewing**	**IQR**	**P-value with the Wilcoxon Rank Sum Test**	**Initial follow-up Parameter estimate (*P*-Value)**	**Day 365 *P*-Value**	**Day 730 *P*-Value**	**Time Effect (*P*-Value)**
Late dysphagia	7	50	(50.0, 100.0)	0.3690	−0.25 (0.0107)	0.2233	0.0394	−0.0016 (0.0013)
No late dysphagia	39	50	(50.0, 100.0)					
**Grade ≥3 Toxicity**	***n***	**Median baseline Swallowing**	**IQR**	**P-value with the Wilcoxon Rank Sum Test**	**Initial follow-up Parameter estimate (*****P*****-Value)**	**Day 365** ***P*****-Value**	**Day 730** ***P*****-Value**	**Time Effect (*****P*****-Value)**
Late dysphagia	7	67	(67.0, 67.0)	0.0253	−2.56 (0.0189)	0.0128	0.0172	−0.0014 (0.0004)
No late dysphagia	39	67	(67.0, 100.0)					
**Grade ≥3 Toxicity**	***n***	**Median baseline Appearance**	**Interquartile Range**	**P-value with the Wilcoxon Rank Sum Test**	**Initial follow-up Parameter estimate (*****P*****-Value)**	**Day 365** ***P*****-Value**	**Day 730** ***P*****-Value**	**Time Effect (*****P*****-Value)**
Late skin toxicity	9	75	(50.0, 75.0)	0.3233	−1.57 (0.0777)	0.1077	0.2260	−0.00035 (0.3347)
No late skin toxicity	39	75	(50.0, 100.0)					
**Grade ≥3 Toxicity**	***n***	**Median baseline Saliva**	**Interquartile Range**	***P*****-value with the Wilcoxon Rank Sum Test**	**Initial follow-up Parameter estimate (*****P*****-Value)**	**Day 365** ***P*****-Value**	**Day 730** ***P*****-Value**	**Time Effect (*****P*****-Value)**
Late xerostomia	7	67	(33.0, 100.0)	0.6376	−1.59 (0.3311)	0.1986	0.1324	0.00065 (0.1363)
No late xerostomia	40	67	(33.0, 100.0)					
**Grade ≥3 Toxicity**	***n***	**Median baseline Taste**	**Interquartile Range**	***P*****-value with the Wilcoxon Rank Sum Test**	**Initial follow-up Parameter estimate (*****P*****-Value)**	**Day 365** ***P*****-Value**	**Day 730** ***P*****-Value**	**Time Effect (*****P*****-Value)**
Late xerostomia	7	33	(33.0, 67.0)	0.1488	−1.75 (0.0512)	0.1629	0.5586	−0.00048 (0.1272)
No late xerostomia	41	67	(33.0, 100.0)					
**Grade ≥3 Toxicity**	***n***	**Median baseline Activity**	**IQR**	***P*****-value with the Wilcoxon Rank Sum Test**	**Initial follow-up Parameter estimate (*****P*****-Value)**	**Day 365** ***P*****-Value**	**Day 730** ***P*****-Value**	**Time Effect (*****P*****-Value)**
Late fatigue	6	50	(50.0, 75.0)	0.2827	0.51 (0.5919)	0.2749	0.0298	−0.00046 (0.1300)
No late fatigue	41	75	(50.0, 75.0)					
**Grade ≥3 Toxicity**	***n***	**Median baseline Mood**	**IQR**	***P-*****value with the Wilcoxon Rank Sum Test**	**Initial follow-up Parameter estimate (*****P*****-Value)**	**Day 365** ***P*****-Value**	**Day 730** ***P*****-Value**	**Time Effect (*****P*****-Value)**
Late fatigue	6	25	(25.0, 75.0)	0.0677	−1.03 (0.2760)	0.2141	0.3179	−0.00054 (0.0724)
No late fatigue	42	75	(50.0, 75.0)					
**Grade ≥3 Toxicity**	***n***	**Median baseline Pain**	**IQR**	***P-*****value with the Wilcoxon Rank Sum Test**	**Initial follow-up Parameter estimate (*****P*****-Value)**	**Day 365** ***P*****-Value**	**Day 730** ***P*****-Value**	**Time Effect (*****P*****-Value)**
Late osteonecrosis	13	50	(50.0, 75.0)	0.4901	−2.14 (0.0026)	0.0536	0.7104	−0.00107 (0.0024)
No late osteonecrosis	35	75	(50.0, 100.0)					

*QOL, Quality of life; IQR, Interquartile range; WRST, Wilcoxon Rank-Sum Test; ECOG, Eastern Cooperative Oncology Group; KPS, Karnofsky Performance Status*.

Patients with late osteonecrosis had similar baseline pain PR-QOL (*p* = 0.49) but significant differences post-treatment at initial follow-up (*p* = 0.0026). This difference trended toward significance at day 365 (*p* = 0.054) but not at day 730 (*p* = 0.71). Late skin toxicity had no significant effect on appearance PR-QOL (*p* = 0.078). Late xerostomia had no significant effect on saliva PR-QOL (*p* = 0.33) but trended toward significant for taste (*p* = 0.051). Late fatigue had no significant effect on activity PR-QOL (*p* = 0.59) or mood (*p* = 0.28).

## Discussion

This is the first study reporting PR-QOL outcomes following re-irradiation with SBRT for rHNC in patients with >1 year of PR-QOL follow-up. Given the poor prognosis of this disease, our sample size is relatively small. However, we emphasize that this is the largest cohort thus reported with long-term outcomes in this population.

Consistent with previous investigations of SBRT re-irradiation (7), our cohort demonstrated a brief decline in PR-QOL across most surveyed domains immediately post-treatment, followed by a gradual return to baseline levels or beyond by 1 year. Similar findings were also reported by Chen et al. in an investigation of functional status and QOL 1 year after IMRT re-irradiation for recurrent head and neck cancer (15). Our long-term (>1 year) follow-up, however, revealed a subsequent decline in PR-QOL across virtually all domains, and overall PR-QOL was significantly worse than baseline beyond day 365 post-treatment. The cause of this decline is not clear, although we postulate that it may possibly be related to disease progression vs. treatment-related toxicities.

Our results show that patients with grade ≥3 late toxicities experience significantly reduced quality-of-life, underscoring the need for early identification of patients at risk for severe late toxicities. The most prevalent grade ≥3 late toxicities in our cohort were dysphagia (*n* = 10, 16%) and osteonecrosis (*n* = 7, 11%), consistent with the literature on the most common late toxicities following re-irradiation with SBRT for rHNC (9). Moreover, severe dysphagia predicted decrements in PR-QOL in chewing and swallowing post-treatment. Severe late osteonecrosis predicted significantly worse pain at initial follow-up, but this difference become non-significant with additional follow-up.

Prior work suggests that the overall risk of developing grade ≥3 late toxicity appears to be associated with the site of recurrence. Ling et al. report that re-irradiation with SBRT to the larynx or hypopharynx is significantly more likely to result in severe late toxicity, compared to treatment of other sites of recurrence within the head and neck. Rates of grade ≥3 late toxicity were observed in 50% of patients who were treated to the larynx or hypopharynx, compared to 6–20% for other sites (9). Given our observed association between grade ≥3 late toxicity and worsened overall PR-QOL, we recommend adherence to dose-volume considerations when selecting patients with recurrence in the larynx or hypopharynx for treatment.

Our observed association between tumor volume >25 cc and worsened overall PR-QOL is consistent with existing literature. Kodani et al. reported improved overall survival among patients with smaller tumors receiving SBRT re-irradiation for rHNC (16), and Vargo et al. demonstrated better local control in patients with tumors <25 cc receiving re-irradiation with SBRT for non-squamous cell cancers of the head and neck (10).

Site of recurrence also predicted specific changes in PR-QOL domains. Patients with oral cavity recurrence had significantly lower chewing PR-QOL, but these patients also had significantly different baseline chewing PR-QOL. Taste, however, was similar at baseline with significant differences at 1- and 2-year post-treatment. However, there were no significant differences in swallowing or speech PR-QOL over time in these patients. Nasal recurrence predicted significant decline in taste. To our surprise, salivary gland recurrence did not significantly affect PR-QOL in chewing, swallowing, taste, or saliva. Moreover, while local failure accounts for the majority of morbidity and mortality in this disease, we did not observe any significant differences in post-treatment PR-QOL with respect to local, nodal, or distant failure.

Our data showed no significant differences in overall PR-QOL with respect to age >65. This finding is noteworthy because patients over 65–70 years of age are frequently excluded from trials that guide clinical management (17). Moreover, our data adds to the conclusions made by Vargo et al. in a prospective study of 12 elderly patients receiving SBRT for primary treatment of medically-inoperable head and neck cancer (18). This study found that SBRT was well-tolerated among the elderly for primary treatment of head and neck cancer. Therefore, we believe SBRT offers encouraging results in this cohort as a means of local disease control while preserving long-term overall PR-QOL.

Our study has several limitations. First, while our median PR-QOL follow-up was 21 months, we had relatively few surveys at 24 months post-treatment and beyond. Two hundred sixty (85%) of the surveys were completed between baseline and 18 months, compared to 19 (6%) at 24 months, 13 (4%) at 36 months, and 14 (5%) at 48 months. This distribution of follow-up times, however, appears to be consistent with the natural progression of the disease, as previous studies in this cohort have reported a median time to death or last clinical follow-up of 10 months (9). Again, we emphasize that this is the largest cohort thus studied of late survivors of recurrent head and neck cancer treated with SBRT, and we believe these findings will guide future management of patients with this disease.

## Conclusions

At 1-year post-treatment, re-irradiation with SBRT for rHNC preserves PR-QOL across multiple domains. However, global decrements in PR-QOL occur with long-term follow-up. Additional vigilance should be exercised in identifying patients at risk for grade ≥3 late toxicity, which is a predictor for worse overall PR-QOL.

## Data Availability Statement

The datasets for this article are not publicly available because of patient confidentiality. Requests to access the datasets should be directed to Dwight Heron, MD, MBA, FACRO, FACR: heronde@icloud.com.

## Ethics Statement

This study was reviewed and approved by the University of Pittsburgh Institutional Review Board, IRB # PRO13020306.

## Author Contributions

Study concept and design were proposed by DH. Data acquisition and manuscript preparation was performed by JT. Data analysis and interpretation were performed by JT and HW. All authors were involved with manuscript editing, review, and involved with quality control of data.

### Conflict of Interest

The authors declare that the research was conducted in the absence of any commercial or financial relationships that could be construed as a potential conflict of interest.
